# 2019 novel coronavirus disease with secondary ischemic stroke: two case reports

**DOI:** 10.1186/s12883-020-02033-3

**Published:** 2021-01-05

**Authors:** Bin Fu, Yun Chen, Ping Li

**Affiliations:** Department of Neurology, Hubei Provincial Hospital of Integrated Chinese and Western Medicine, Hubei University of Chinese Medicine, 430000 Wuhan, China

**Keywords:** Coronavirus disease-19 (COVID-19), Severe acute respiratory syndrome coronavirus 2 (SARS-CoV-2), Secondary ischemic stroke

## Abstract

**Background:**

The COVID-19 pandemic, which broke out in Wuhan in 2019, has become the global health crisis of our time. Elderly patients with certain fundamental diseases are more likely to develop severe cases. The secondary lesion following viral infection have only rarely been reported.

**Case presentation:**

We here report two cases of coronavirus-infected pneumonia with acute ischemic stroke in middle-aged patients. In both COVID-19 cases, neurological physical examinations showed normal results before infection. Lymphocytopenia, accompanied by elevated cytokines and D-dimers, were found from serum clinical laboratory examination at admission. Dysarthria and limb muscle weakness are initial manifestations, occurring one week after infect-causative pathogen, SARS-CoV-2. The head CT and head/neck arterial CTA showed small-vessel occlusion. The patients were diagnosed with coronavirus diseases with secondary acute ischemic stroke. They were treated with tirofiban and followed up with daily aspirin and atorvastatin.

**Conclusions:**

These cases suggested that secondary ischemic stroke, mainly manifested as small-vessel occlusion, should be considered for COVID-19 patients and diagnosed and treated promptly.

## Background

At the end of 2019, a novel type of coronavirus disease (COVID-19) broke out in Wuhan, China. The global number of confirmed cases of COVID-19 is still increasing daily. The main clinical manifestations of this disease are lung symptoms such as fever, cough, and wheezing [1], [2]. Patients with concomitant diseases are more likely to develop severe or critical cases and die from acute respiratory distress. Ling et al. found that 36% of hospitalized patients had neurological manifestations like headaches and dizziness [3]. Another large-scale epidemiologic study showed that 1.5% of the COVID-19 patients had histories of cerebrovascular disease [1]. Nevertheless, secondary lesions following infection have only rarely reported. Here we present case reports of two confirmed middle-aged patients of COVID-19 with acute ischemic stroke. These two patients received intensive care in the isolation ward and were recently discharged from the hospital. The written consent was obtained from both patients in the consent for publication statement.

## Case presentation

### Case 1

**Fig. 1:**
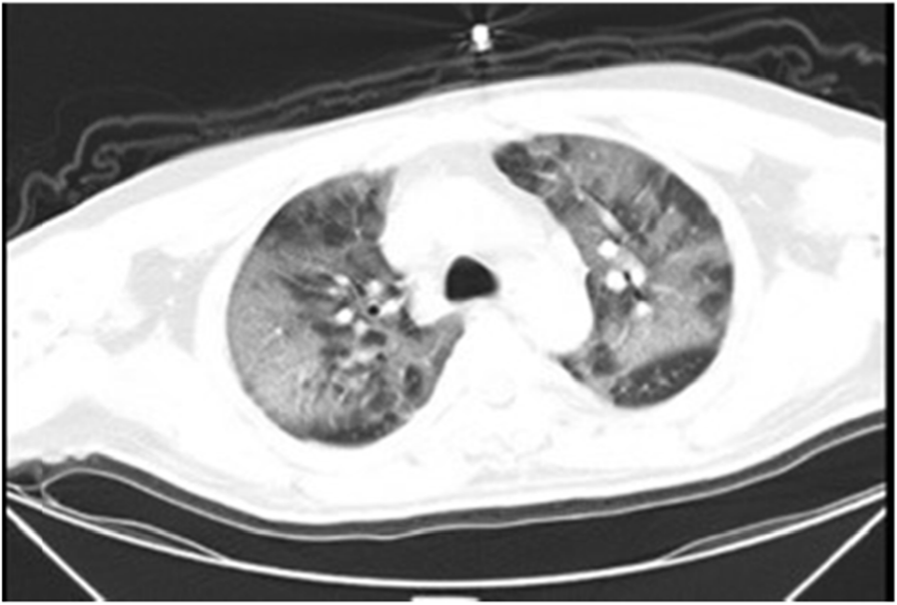
CT imaging of case 1 infected with SARS-CoV-2 on January 5, 2020, Illness day 7

A 45-year-old man was admitted for fever over one week on January 5, 2020. The vital signs from a general physical examination were 38.3 °C body temperature, 110 times/min for pulse, 24 times/min for breathing rate, and 125/72 mmHg for blood pressure. The patient reported no history of hypertension, diabetes, hyperlipidemia, or cardiac disease, and no history of smoking or drinking. His neurological examination on admission was normal. The clinical serum laboratory examination showed an increase in serum amyloid protein (SAA) of 288.42 mg/L (range 0–10 mg/L), C reactive protein (CRP) of 25.17 mg/L (range 0–10 mg/L), D-dimer of 15.21 mg/L (range 0-0.5 mg/L) and fibrinogen (FIB) of 4.87 G/L (range 2–4 G/L). The levels of serum cytokines were also profoundly increased: IL-8 increased to 438.20 pg/ml (range 0–62 pg/ml), IL-10 increased to 836.50 pg/ml (range 0-9.1 pg/ml), and IL-6 increased to 962.70 pg/ml (range 0–7 pg/ml). The WBC was normal together with lymphocytopenia (0.64 G/L, range 0.8-4 G/L). The levels of glucose, lipids, and homocysteine are normal. Typical bilateral patchy shadowing was observed on chest computed tomography (CT) scans (Fig. 1). The real-time RT-PCR assay for the SARS-CoV-2 test was positive.

**Fig. 2:**
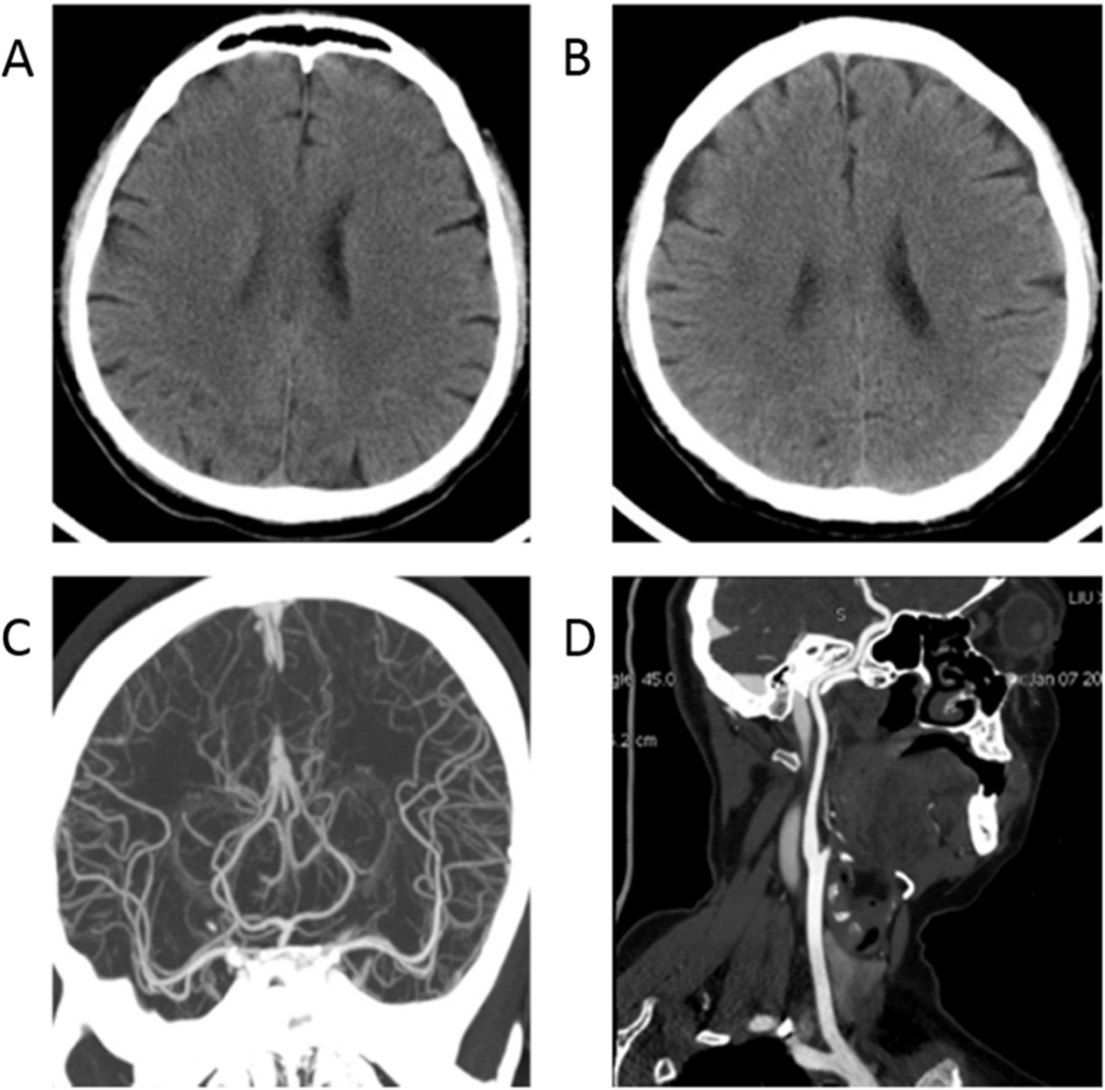
The head CT and head/neck arterial CTA of case 1 infected with SARS-CoV-2. **a**: Head CT on January 6, 2020, Illness day 8, **b**: Repeat head CT on January 8, 2020, Illness day 10; **c**: Head arterial CTA on January 6, 2020, Illness day 8, **d**: Neck arterial CTA on January 6, 2020, Illness day 8

The patient was treated with oxygen therapy, ribavirin (0.5 g, i.v. drip, Q12h), and venous rehydration solution. At 9:00 a.m. on January 6, the patient was found to have stroke symptoms, such as unclear speech, weakness of the left limb (4/5 muscle strength throughout), shallow left nasolabial groove, and tongue toward to the left. Muscle strength declined to 1/5 of the upper limb and 2/5 of lower limbs at 5:00 p.m. The NIHSS score was 9. The laboratory examination showed increased levels of D-dimer (32 mg/L) and decreased levels of FIB (1.06 G/L), with longer prothrombin time (PT, 14.7 s, range 11–14 s) and thrombin time (TT, 22.7 s, range 9–20 s). However, the head CT (Fig. 2a) and neck arterial CTA (Fig. 2c and d) did not show any apparent abnormalities at that time. The patient was treated with atorvastatin (20 mg, p.o., q.d.), tirofiban (0.1 ug/kg.min, continuous intravenous pumping for 48 hours), following by daily aspirin (100 mg, p.o.) and clopidogrel (75 mg, p.o.). On January 8, neurological function defects about dysarthria and left limb weakness of the patient got recovery gradually. Repeat head CT examination two days later showed infarction of the right corona radiata (Fig. 2b). The patient did not have a fever in the later of hospitalization, and his pulmonary symptoms were significantly improved. The patient was discharged from the hospital on February 3 with mild dysarthria. His left limb muscle strength score reached 5. The NIHSS score declined to 2 from 9, and the MRS score was 1 on the discharge day.

### Case 2

**Fig. 3:**
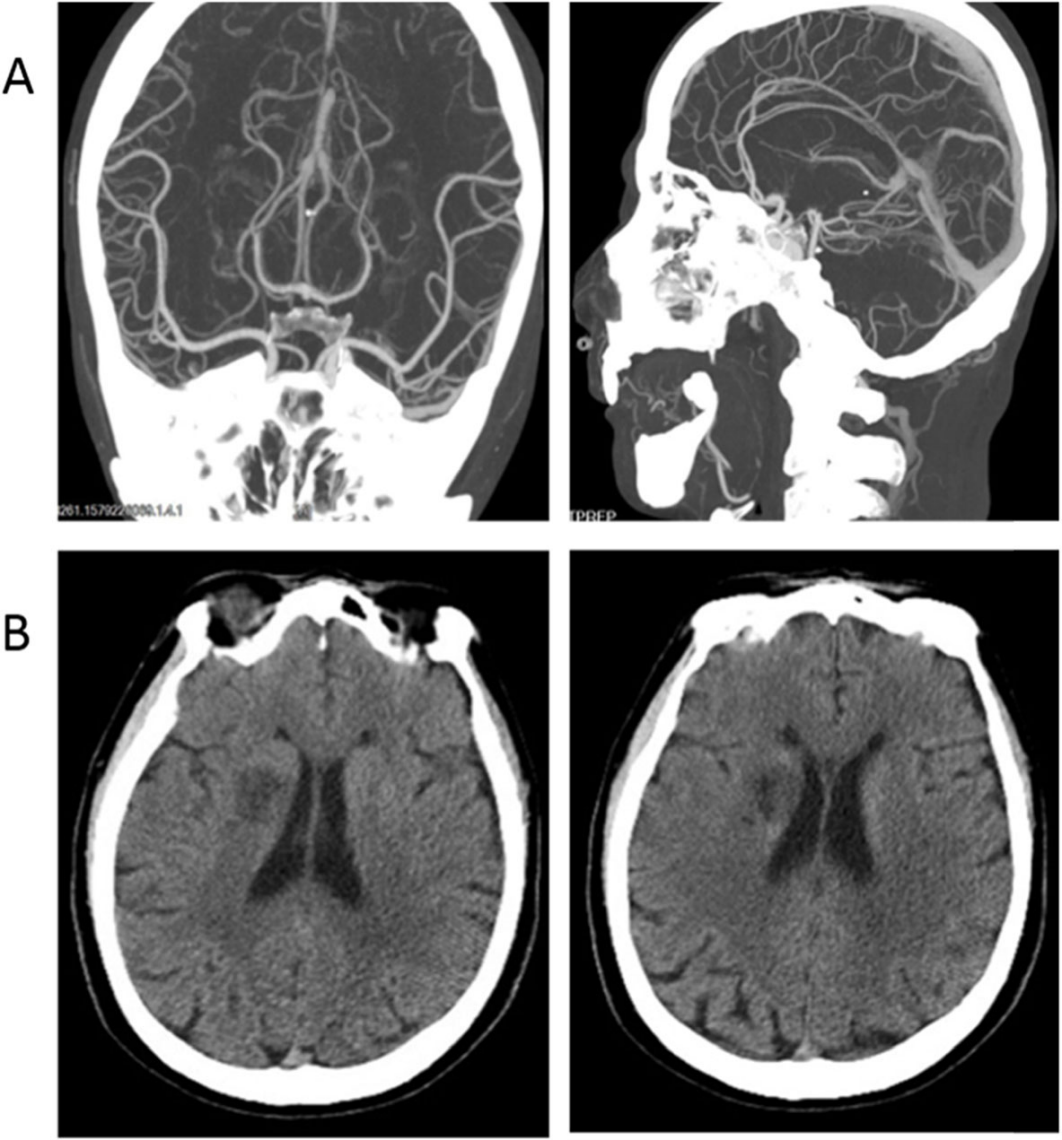
The head/neck atrial CTA and head CT of case 2 infected with SARS-CoV-2. **a**: Head CTA on January 17, 2020, routine surgical evaluation before thyroid cancer removal surgery, **b**: Head CT on February 10, 2020, Illness day 9

**Fig. 4:**
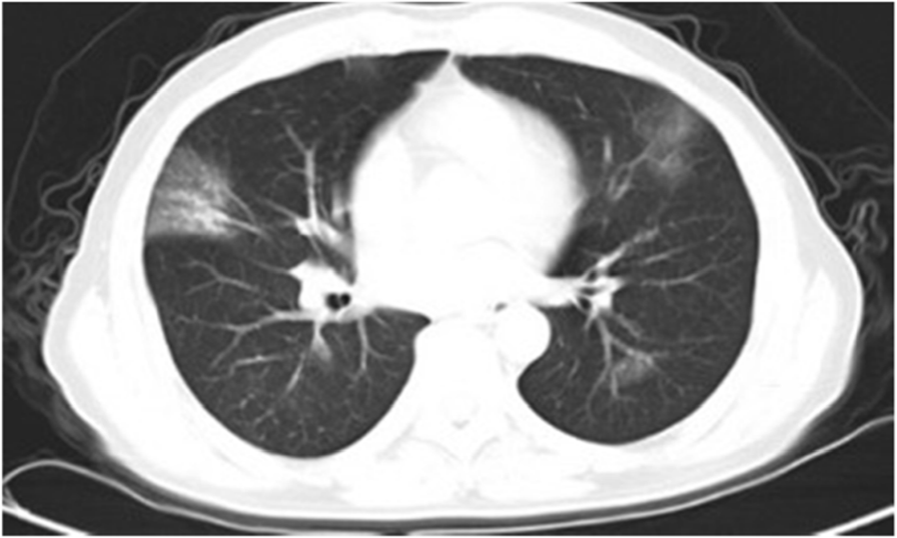
CT imaging of case 2 infected with SARS-CoV-2 on February10, 2020, Illness day 9

A 50-year-old man was admitted on February 10 for fever lasting nine days with a sudden left limb weakness lasting 28 hours. The patient had a 20-years history of smoking and did not have any history of hypertension, diabetes, hyperlipidemia, or cardiac diseases. He had undergone surgery to remove a cancerous mass from his left thyroid on January 19, 2020 in our hospital and had been discharged after recovery. The routine surgical evaluation on January 17 did not find any abnormalities from the color-Doppler ultrasound and CT angiography examinations except for basilar artery fenestration (Fig. 3a). The general physical examination at admission on February 10 was 37.8 °C for body temperature, 78 times/min for pulse, 18 times/min for breathing rate, and 132/76 mmHg for blood pressure. Neurological examinations showed shallow left nasolabial grooves, tongue toward left and dysarthria, left upper limb muscle strength score 3, and a positive reflex of Babinski’s sign. The NIHSS score was 7. Laboratory examination displayed increased levels of SAA (> 300 mg/L), CRP (93.15 mg/L), D-dimer (19.86 mg/L), longer PT (15.4 s) and lymphocytopenia (0.39 G/L). The levels of serum cytokines, such as IL-8 of 84.90 pg/ml, IL-1β of 36.60 pg/ml (range 0–5 pg/ml) and IL-10 of 26.70 pg/ml (range 0-9.1 pg/ml) were increased. The levels of glucose, lipids, and homocysteine were normal. The COVID-19 was diagnosed from typical bilateral patchy shadowing from chest CT (Fig. 4) and positive RT-PCR assay test. Head CT performed on February 10 presented right basal ganglia infarction (Fig. 3b). Then the patient was treated with oxygen therapy, ribavirin (0.5 g, i.v. drip, Q12h), and venous rehydration solution, following by daily aspirin (100 mg, p.o.), clopidogrel (75 mg, p.o.) and atorvastatin (20 mg, p.o.). During hospitalization, the patient’s fever ceased and did not recur, and the pulmonary symptoms were markedly relieved. However, defects in neurological function recovered only gradually. The patient was discharged on March 1 with mild dysarthria. The left limb muscle strength score was 4, the NIHSS score was 5, and the MRS score was 3.

## Discussion and conclusion

We report two cases of COVID-19 in patients with acute ischemic stroke. Previous studies have found that multiple organs other than the lung were also involved in COVID-19 [4]. Recently Hu et al. reported that neurological symptoms developed in one-third of hospitalized patients [3]. Other studies of coronavirus induced diseases (such as SARS [5], MERS [6]) also involved cases of central nervous system damage.

For both patients, neurological physical examination showed normal results before SARS-CoV-2 infection. Our initial speculation for this acute ischemic stroke is a secondary lesion one week after COVID-19 onset. The possible mechanism might involve the way that the coronavirus attacks the human body through the angiotensin-converting enzyme-2 [7] which is distributed on blood vessels and multiple organs [8]. The virus triggers a cytokine cascade that could aggravate ischemic brain damage and increase the risk of intracerebral hemorrhage [9] and blood coagulation disturbances. This hypothesis is supported by the high expression of cytokines and D-dimers observed in these two patients. Recently, many works have shown that the virus can cause thrombosis in the lungs and subsequent multi-organ failure [10]. Even the biological mechanisms underlying the elevated level of plasma D-dimers in COVID-19 patients are unclear [11]. Thachil et al. suggested that low molecular weight heparin (LMWH) at prophylactic doses should be beneficial for patients with markedly elevated D-dimers [12]. Notably, these two patients were middle-aged and should had a low risk of stroke. They did not show any symptoms of deep venous thrombosis or large vessel occlusion with endoluminal thrombosis. We suspected that the small-vessel occlusion was secondary lesion following coagulopathy during SARS-CoV-2 infection. Unfortunately, we could not have more exhaustive neurological examinations during this hospitalization because of the exceptional infectious circumstances. Thus, there are still some concerns about increased D-dimer levels and ischemic stroke or thrombosis in multiple organs from a clinical perspective.

In case 1, we addressed the possibility of small-vessel occlusion developing into progressive stroke, using tirofiban instead of aspirin and clopidogrel. We did so with the informed consent of the patient and acted in accordance with the Chinese expert consensus on the clinical use of tirofiban in atherosclerotic cerebrovascular diseases [13] and guidelines for the early management of patients with acute ischemic stroke [14]. The patient’s neurological function recovered rapidly over 2 days. Such platelet glycoprotein II.b/III.a receptor antagonists could act on the final common channel of thrombosis. Their efficacy was found to have no significant difference from aspirin in the incidence of symptomatic intracranial hemorrhage [15, 16]. It is important to note that the LMWH can reduce the risk of thromboembolism in our case with hypomobility according to the new consensus [17].

These are the only two reported cases of COVID-19 with secondary acute ischemic stroke out of 764 hospitalized patients in Hubei Provincial Hospital of Integrated Chinese and Western Medicine until March 9, 2020. We suggest early diagnosis and treatment should be considered for COVID-19 patients with secondary ischemic stroke, which manifests mainly as small-vessel occlusion.

## Data Availability

No datasets were analyzed during the current study.

## Abbreviations

COVID-19: Coronavirus disease-19
SARS-CoV-2: Severe acute respiratory syndrome coronavirus 2
CT: Computed tomography
CTA: Computerized tomography angiography
RT-PCR: Reverse transcription-polymerase chain reaction
SAA: Serum amyloid protein
FIB: Fibrinogen
CRP: C reactive protein
PT: Prothrombin time
TT: Thrombin time
NIHSS: National Institutes of Health Stroke Scale
MRS: Modified Rankin Score
WBC: White blood cell
